# IHBOFS: A Biomimetics-Inspired Hybrid Breeding Optimization Algorithm for High-Dimensional Feature Selection

**DOI:** 10.3390/biomimetics11010003

**Published:** 2025-12-22

**Authors:** Chunli Xiang, Jing Zhou, Wen Zhou

**Affiliations:** 1 School of Computer Science, Hubei University of Technology, No. 28 Nanli Road, Hongshan District, Wuhan 430068, China; xiangchunli@hbut.edu.cn (C.X.); zhoujing2024@hbut.edu.cn (J.Z.); 2 Hubei Provincial Key Laboratory of Green Intelligent Computing Power Network, No. 28 Nanli Road, Hongshan District, Wuhan 430068, China

**Keywords:** high-dimensional feature selection, hybrid breeding optimization, elite opposition-based learning, good point set

## Abstract

With the explosive growth of data across various fields, effective data preprocessing has become increasingly critical. Evolutionary and swarm intelligence algorithms have shown considerable potential in feature selection. However, their performance often deteriorates in large-scale problems, due to premature convergence and limited exploration ability. To address these limitations, this paper proposes an algorithm named IHBOFS, a biomimetics-inspired optimization framework that integrates multiple adaptive strategies to enhance performance and stability. The introduction of the Good Point Set and Elite Opposition-Based Learning mechanisms provides the population with a well-distributed and diverse initialization. Furthermore, adaptive exploitation–exploration balancing strategies are designed for each subpopulation, effectively mitigating premature convergence. Extensive ablation studies on the CEC2022 benchmark functions verify the effectiveness of these strategies. Considering the discrete nature of feature selection, IHBOFS is further extended with continuous-to-discrete mapping functions and applied to six real-world datasets. Comparative experiments against nine metaheuristic-based methods, including Harris Hawk Optimization (HHO) and Ant Colony Optimization (ACO), demonstrate that IHBOFS achieves an average classification accuracy of 92.57%, confirming its superiority and robustness in high-dimensional feature selection tasks.

## 1. Introduction

With the rapid expansion of dataset dimensions, high-dimensional data pose numerous challenges. In such spaces, data become sparse, making it difficult for models to learn effective patterns, which reduces the generalization capability [1]. When models contain excessive or redundant features, they tend to capture irrelevant patterns from the training samples, which in turn degrades their generalization ability on new data [2]. Moreover, as the dimensionality increases, the number of potential feature combinations grows exponentially, causing traditional search-based selection procedures to become computationally prohibitive [3]. Therefore, recent research has increasingly turned toward adaptive and scalable feature reduction techniques designed for complex high-dimensional datasets [4].

Feature selection (FS) serves as a fundamental procedure in the fields of machine learning and data analytics. Its objective is to extract the most informative subset of attributes from the full feature set, thereby enhancing model efficiency, reducing the computational burden, and improving the interpretability of learning outcomes [5]. Broadly, FS methods can be divided into three major categories: filter-based, wrapper-based, and embedded approaches. The filter-based category assesses feature importance without relying on any particular learning algorithm, often through statistical relevance measures such as correlation analysis, chi-square evaluation, or information gain [6]. Their low computational overhead makes them an effective choice for preprocessing extensive datasets [7]. In wrapper approaches, subsets of features are examined using a designated learning algorithm, training and assessing models to select the optimal subset, though incurring high computational cost [8], with popular approaches like recursive feature elimination (RFE) [9]. Embedded methods integrate FS within the model training procedure by tuning model parameters, allowing for an efficient and effective identification of relevant features [10], and are generally more computationally efficient, often yielding better model performance compared to wrapper methods [11].

Hybrid approaches integrate the advantages of both filter and wrapper techniques. Typically, a preliminary filter step is applied to limit the dimensionality, followed by a wrapper method to refine the optimal feature subset [12]. For example, a recent hybrid feature selection method combines ReliefF and fuzzy entropy with an enhanced equilibrium optimizer and achieves superior performance on medical datasets [13].

Despite the effectiveness of traditional and hybrid feature selection strategies, they often encounter limitations when applied to large-scale or highly complex datasets, particularly owing to the combinatorial complexity of feature subset selection. As the dimensionality of the data increases, exhaustive search becomes computationally infeasible, and heuristic-based methods may fall into local optima. To cope with this challenge, increasing attention has been devoted to evolutionary computation methods, which demonstrate remarkable global exploration ability and adaptability when tackling high-dimensional and nonlinear optimization tasks.

Evolutionary algorithms such as Grey Wolf Optimizer (GWO) [14], Genetic Algorithm (GA) [15], and Particle Swarm Optimization (PSO) [16] simulate natural evolution or swarm intelligence to explore high-quality solutions within challenging optimization landscapes. However, standard metaheuristics tend to converge slowly, with low efficiency in high-dimensional settings. As a result, researchers have considered various approaches to improve the algorithm in order to enhance the feature selection performance [17]. An improved Crayfish Optimization Algorithm (MCOA) was proposed to lead the exploration toward optimal results using an environmental update mechanism [18]. A self-adaptive weighted differential evolution algorithm (SaWDE) was proposed to tackle large-scale feature selection tasks and showed remarkable effectiveness across diverse high-dimensional datasets [19]. The Feature Weighting Particle Swarm Optimization method (FWPSO) effectively identifies biomarker genes within complex microarray data by integrating feature relevance assessment with optimized feature selection [20]. A hybrid Sine Cosine–Firehawk Algorithm (HSCFHA) was designed to perform feature selection by optimizing dataset variance [21]. In the past four years, other methods have been proposed, including VGS-MOEA [22], MTPSO [23], PSO-EMT [24], and DENCA [25].

Despite the effectiveness demonstrated by existing metaheuristic-based feature selection methods, their inherent random strategies, while ensuring global search capability, struggle to completely avoid local optima. Therefore, developing a computationally efficient high-dimensional feature selection method that exhibits good consistency and robustness remains a significant direction in current research. Ye et al. inspired by the theory of hybrid vigor and hybrid breeding mechanisms, proposed a hybrid breeding optimization algorithm [26]. HBO exhibits outstanding performance in exploration, search efficiency, and adaptability and has been employed to address various optimization challenges, including the 0–1 knapsack problem [27]. Despite its novel structure, standard HBO suffers from limited adaptability and may converge prematurely. To overcome these issues, several improved variants have been proposed. For instance, a Cooperative Hybrid Breeding Swarm Intelligence (CHBSI) algorithm was proposed to address the challenges of FS in high-dimensional spaces [28]. Furthermore, a Double-Stage Multimodal HBO (DSMHBO), integrating dynamic niching, neighborhood search, and elite mutation, was proposed to locate multiple optima effectively [29]. In the domain of network security, Ye et al. developed a Cooperative Co-evolution Improved HBO (CCIHBO) framework [30], effectively enhancing intrusion detection accuracy. These improvements significantly broaden the applicability and capability of the original HBO to handle intricate optimization problems involving high-dimensional search spaces.

The performance of HBO has already been validated on function optimization and engineering optimization problems. However, HBO and its improved variants face challenges such as low convergence accuracy and insufficient robustness in high-dimensional FS scenarios. Therefore, this paper focuses on HBO research, with the objective of thoroughly analyzing its potential and limitations in high-dimensional feature selection problems. We propose IHBOFS, which enhances the original HBO framework to better handle high-dimensional feature selection tasks.

The main findings highlighted in this work are as follows:

- An integrated multi-strategy improved hybrid breeding optimization algorithm is proposed, which effectively balances the exploration and exploitation capabilities.
- Ablation experiments are conducted on various types of benchmark functions to verify the effectiveness of the different enhancement strategies incorporated into the improved algorithm.
- The optimal combination of classifier and transfer function is identified based on experimental evaluations of the performance of different options.
- The performance of IHBOFS is evaluated against various metaheuristic-based FS methods on high-dimensional datasets to validate its effectiveness.

The structure of this paper is as follows: Section 2 provides a review of recent related studies, presenting the improvement strategies and their corresponding mathematical models. Section 3 introduces the proposed IHBOFS. Experimental findings and corresponding discussions are detailed in Section 4. Finally, Section 5 concludes the paper and outlines potential directions for future research.

## 2. Related Works

In recent years, numerous evolutionary algorithms have been proposed to address the challenges of high-dimensional feature selection, and attempts have been made to improve algorithm performance from various perspectives [31]. Table 1 summarizes the performance and limitations of several nascent hybrid algorithms tailored for feature selection tasks in various research fields. Among them, the HBO has shown strong potential due to its biological inspiration and excellent global search ability. As this work builds upon HBO as a baseline, this section first introduces the fundamental principles of the HBO and then presents recent improvements and related methods relevant to high-dimensional feature selection.

### 2.1. Hybrid Breeding Optimization Algorithm

To address the inefficiency of FS, Ye et al. [26] proposed the HBO, which exhibits strong exploratory capability and algorithm efficiency. During the initial stage, all individuals are sorted according to their fitness values, forming the population: $X=\{X_{1},X_{2},\ldots ,X_{n}\}$, where *n* represents the population size. The maintainer line, which consists of individuals with the highest fitness values, is defined as $M=\{X_{1},X_{2},X_{3},\ldots ,X_{m}\},\;$m=⌊n/3⌋. The sterile line is composed of individuals exhibiting the poorest fitness $X_{s}=\left\{X_{2m+1},X_{2m+2},\ldots ,X_{n}\right\},$ while the remaining individuals make up the restorer line $X_{r}=\left\{X_{m},X_{m+1},\ldots ,X_{2m}\right\}$.

Hybridization: The sterile individuals are updated during this step. The process of generating new individuals through hybridization is given by Equation (1),

(1) $$\begin{array}{c} X_{s(i)}\left(t+1\right)=k_{1}\cdot X_{s(j)}\left(t\right)+\left(1-k_{1}\right)\cdot X_{m}\left(t\right), \end{array}$$

where $X_{s(i)}\left(t+1\right)$ represents a new sterile-line individual generated in iteration *t*, and $X_{m}\left(t\right)$ and $X_{s(j)}\left(t\right)$ represent individuals randomly drawn from the maintainer and sterile lines, respectively. While $k_{1}$ denotes a uniformly distributed random variable within [0,1], the subsequent parameters $k_{2}$ and $k_{3}$ share the same distribution and are utilized in later stages of the algorithm.

Selfing: In this step, the individuals in the restorer line integrate genetic information from other individuals to facilitate the evolution of the subpopulation towards an optimal solution. This process is formulated as Equation (2),

(2) $$\begin{array}{c} X_{r(i)}\left(t+1\right)=k_{2}\cdot \left(X_{\mathrm{best}}\left(t\right)-X_{r(j)}\left(t\right)\right)+X_{r(i)}\left(t\right). \end{array}$$

In this equation, $X_{r(i)}\left(t+1\right)$ refers to a newly generated individual obtained through self-fertilization between two restorer individuals *i* and *j*. The element $X_{\mathrm{best}}\left(t\right)$ represents the current best individual in iteration *t*, whereas $X_{r(j)}\left(t\right)$ represents another restorer individual randomly chosen from the population.

Renewal: If a restorer-line individual fails to update for a predefined number of consecutive iterations (${\mathrm{SC}}_{\max}$), the algorithm resets it by randomly selecting new values from the search space. This mechanism is described as Equation (3),

(3) $$\begin{array}{c} X_{r(i)}\left(t+1\right)=k_{3}\cdot \left(V_{\max}-V_{\min}\right)+X_{r(i)}\left(t\right)+V_{\min}, \end{array}$$

where $X_{r(i)}\left(t\right)$ represents the restorer individual that has not been updated, and $V_{\max}$ and $V_{\min}$ are the maximum and minimum values of the search space.

### 2.2. Good Point Set

**Figure 1:**
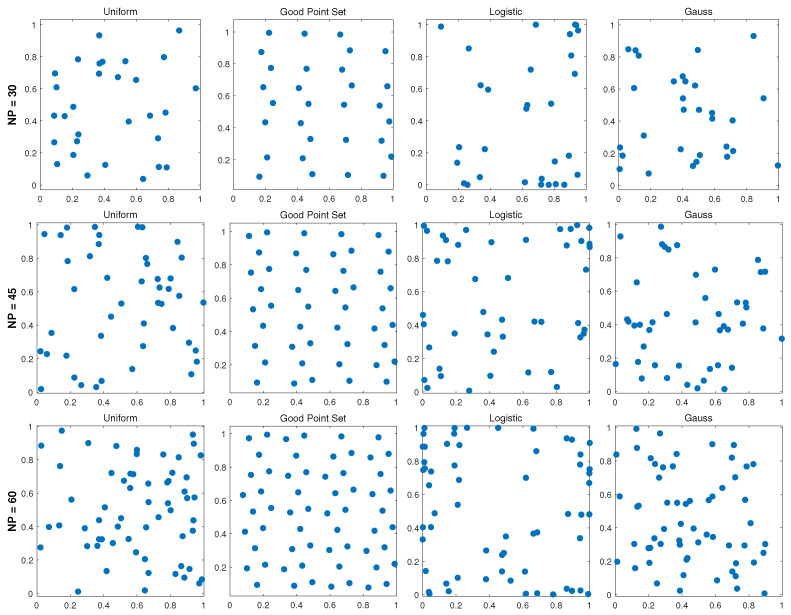
Comparison of population distributions obtained from different initialization approaches.

The Good Point Set (GPS) method, proposed by the Chinese mathematician Luogeng Hua, aims to generate a set of uniformly distributed points within the search space, which helps establish a balanced coverage of the search domain during initialization [41]. Its principle is as follows:

Suppose $G_{D}$ is the unit cube in the *D*-dimensional Euclidean space; then, $P_{n}\left(k\right)=\left(\left\{r_{1}^{n}\cdot k\right\},\left\{r_{2}^{n}\cdot k\right\},\ldots ,\left\{r_{D}^{n}\cdot k\right\}\right)$ is a Good Point Set, where 1≤k≤n and $r\in G_{D}$. The discrepancy is $\varphi \left(n\right)=C\left(r,\varepsilon \right)n^{-1+\varepsilon }$, and C(r,ε) is a constant that only depends on *r* and ε (ε is an arbitrarily small positive number), *r* is the good point, $\left\{r_{s}^{n}\cdot k\right\}$ represents taking the fractional part, and *n* represents the number of points. In this paper, we take $r=2\cos(\frac{2k\pi }{p})$, where 1≤k≤D, and *p* is the smallest prime number satisfying $\frac{p-3}{2}\geq D$. The initialization mapping is defined in Equation (4),

(4) $$\begin{array}{c} X_{i}^{d}=\left({\mathrm{UB}}^{d}-{\mathrm{LB}}^{d}\right)\times \left\{r_{d}^{i}\times k\right\}+{\mathrm{LB}}^{d}, \end{array}$$

where ${\mathrm{LB}}^{d}$ and ${\mathrm{UB}}^{d}$ represent the lower and upper bounds of the *d*-th dimension in the search space, respectively. Figure 1 presents the two-dimensional initial population distributions generated by four different methods—logistic chaotic mapping, Good Point Set initialization, Gaussian perturbation, and uniform random initialization. As depicted, for relatively small population sizes, the Good Point Set-based initialization method significantly improves the uniformity of population distribution. It improves the coverage of the search space, reducing the sensitivity to the initial population distribution.

### 2.3. Elite Opposition-Based Learning (EOBL)

The core idea of EOBL is to exploit the symmetry of the problem space by mirroring each solution in the representation space. The principle is as follows:

Consider an elite individual $X_{i}=\left(x_{i,1},x_{i,2},\ldots ,x_{i,D}\right)$, where i=1,2,…,n, in a *D*-dimensional search space. Its opposite solution is defined as Equations (5) and (6):

(5) $${\tilde{X}}_{i}=\left({\tilde{x}}_{i,1},{\tilde{x}}_{i,2},\ldots ,{\tilde{x}}_{i,D}\right),$$

(6) $${\tilde{x}}_{i,j}=K\left({\mathrm{LB}}_{j}+{\mathrm{UB}}_{j}\right)-x_{i,j},\;j=1,2,\ldots ,D,$$

where *K* is a dynamic coefficient taking values in [0, 1], and $LB_{j}$ and $UB_{j}$ denote the lower and upper bounds of the *j*-th dimension, respectively. When the computed opposite solution falls outside the search boundaries, it is adjusted according to a uniform distribution, as described in Equation (7).

(7) $${\tilde{x}}_{i,j}=\mathrm{Uniform}\left({\mathrm{LB}}_{j},{\mathrm{UB}}_{j}\right)$$

The standard HBO mainly utilizes heterosis to update sterile lines and restorer lines, thereby achieving population evolution. However, the maintainer line lacks an effective update strategy, leading to underutilization. In addition, the original operators exhibit performance deficiencies in high-dimensional complex scenarios. With the aim of resolving these limitations, we designed four advanced strategies to optimize and improve the standard HBO.

## 3. The Proposed Method

A thorough explanation of the proposed method is provided in this section.

### 3.1. Integrated Multi-Strategy Improved HBO

The subsequent discussion offers a comprehensive introduction to the algorithm’s process and improvement measures.

#### 3.1.1. Optimization of Initial Population Generation

Initial population generation is a crucial step in metaheuristic algorithms, directly affecting the search efficiency and accuracy. In the traditional HBO, random initialization is used. However, an excessively large population increases the computational burden, whereas a too-small population leads to uneven distribution, making it susceptible to becoming trapped in local optima, particularly for complex high-dimensional problems. Therefore, this paper introduces the GPS and EOBL to optimize the initial population generation. The GPS-initialized population is used to construct elite opposition solutions, from which the best individuals are selected as the final initial solution set.

#### 3.1.2. Operations for the Maintainer Line

The sterile line holds significant potential. When guided in evolution by maintainer lines with currently high fitness values, there is a considerable probability of generating superior individuals. However, due to the low stability of the sterile line, strategy adjustments are required at different stages of the iteration process. Therefore, three refined variants of differential operators are introduced in this paper, each designed to operate at different stages of the search process. These operators, denoted as $DE_{1}$, $DE_{2}$, and $DE_{3}$, are mathematically defined in the following Equations (8)–(10).

(8) $$\begin{array}{cc} {\mathrm{DE}}_{1}:X_{i}\left(t+1\right) & =X_{i}\left(t\right)+r_{1}\cdot \left(X_{m(i)}\left(t\right)-X_{m(j)}\left(t\right)\right)+\left(1-r_{1}\right)\cdot \left(X_{m(k)}\left(t\right)-X_{i}\left(t\right)\right), \end{array}$$

(9) $$\begin{array}{cc} {\mathrm{DE}}_{2}:X_{i}\left(t+1\right) & =X_{i}\left(t\right)+F\cdot \left(X_{m(i)}\left(t\right)-X_{m(j)}\left(t\right)\right)+F\cdot \left(X_{\mathrm{best}}\left(t\right)-X_{i}\left(t\right)\right), \end{array}$$

(10) $$\begin{array}{cc} {\mathrm{DE}}_{3}:X_{i}\left(t+1\right) & =X_{\mathrm{best}}\left(t\right)+r_{2}\cdot \left(X_{m(i)}\left(t\right)-X_{m(j)}\left(t\right)\right)+\left(1-r_{2}\right)\cdot \left(X_{m(k)}\left(t\right)-X_{m(l)}\left(t\right)\right), \end{array}$$

where $X_{\mathrm{best}}\left(t\right)$ indicates the optimal solution obtained at iteration *t*, and $X_{m(\cdot )}$ refers to individuals randomly drawn from the maintainer line, with all indices being distinct. The updated value of the *i*-th maintainer line individual at iteration t+1 is represented by $X_{i}\left(t+1\right)$. The smoothing factor *F* controls the transition from a global to a local search, and the random coefficients $r_{1}$ and $r_{2}$ are independently generated within the interval [0, 1].

To allow different differential operators to be applied with varying probabilities during different phases of optimization, the following strategy is implemented: at the initial stage, the global differential operator ${\mathrm{DE}}_{1}$ is assigned a greater likelihood of selection, whereas during the the global-to-local search transition, the transition operator ${\mathrm{DE}}_{2}$ is preferred. At the later stage, the local differential operator ${\mathrm{DE}}_{3}$ is preferred to further refine the optimal solution. An adaptive algorithm is proposed to dynamically determine the selection probabilities corresponding to the three differential operators, while utilizing a roulette wheel selection algorithm for operator selection, as shown in Equations (11)–(17):

(11) $$s_{1}=k\cdot \frac{1}{1+e^{\left(t-T/6)/(0.04*T\right)}}+s_{\min},$$

(12) $$S_{2}\left(t\right)=k\cdot e^{-{\left(t-T/2\right)}^{2}/\left(10\cdot T\right)}+S_{\min},$$

(13) $$S_{3}\left(t\right)=k\cdot \frac{1}{1+e^{-(t-5T/6)/(0.04T)}}+S_{\min},$$

(14) $$k=s_{\max}-s_{\min},$$

(15) $$S=s_{1}+s_{2}+s_{3},$$

(16) $$p_{i}=\frac{s_{i}}{S},\;i=1,2,3,$$

(17) $${\mathrm{DE}}_{s}=\mathrm{RW}\text{\_}\mathrm{Select}\left(p_{1},p_{2},p_{3}\right),$$

where $s_{\min}$ and $s_{\max}$ are parameters to constrain the lower and upper bounds before normalization. $P_{i}$ denotes the probability for selecting each differential operator after normalization. Here, *t* and *T* denote the current and maximum iteration counts, respectively. $DE_{s}$ corresponds to the differential operator finally selected via the roulette wheel strategy.

**Figure 2:**
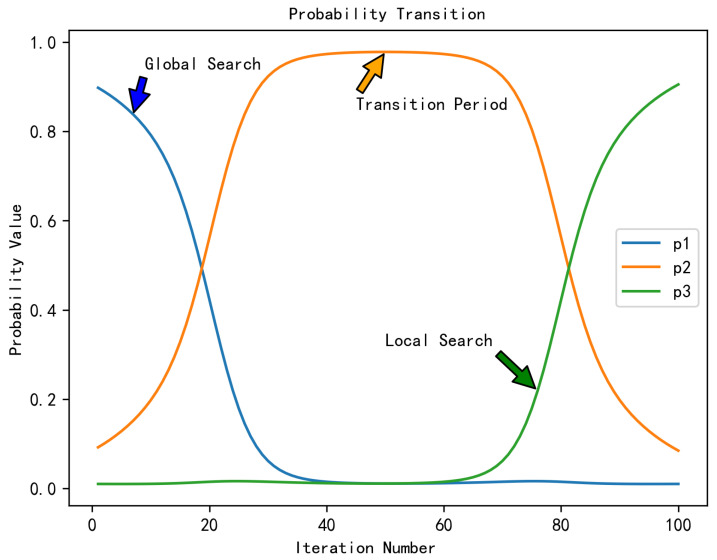
The evolving selection chances of the three differential operators over successive iterations.

As shown in Figure 2, the algorithm adjusts the selection probabilities of each operator as the iteration progresses.

#### 3.1.3. Operations for the Hybridization Phase

To strengthen the hybridization process, a *t*-distribution mutation-based perturbation method is adopted. The modification of the HBO crossover phase is expressed in Equations (18)–(21):

(18) $$X_{s(i)}\left(t+1\right)=t\cdot X_{s(j)}\left(t\right)+\left(1-t\right)\cdot X_{m}\left(t\right),\;i\neq j,$$

(19) $$f\left(t|df,\sigma \right)=\frac{\Gamma \left(\frac{df+1}{2}\right)}{\Gamma \left(\frac{df}{2}\right)\sqrt{df\pi }\sigma }{\left(1+\frac{1}{df}{\left(\frac{t}{\sigma }\right)}^{2}\right)}^{-\frac{df+1}{2}},$$

(20) $$df=2+\frac{t}{T}\cdot 28,$$

(21) $$\sigma =\left(\sigma_{\max}-\sigma_{\min}\right)\cdot \left(1-{\left(\frac{t}{T}\right)}^{gr}\right)+\sigma_{\min}.$$

The original random number *r* is replaced by a value selected through a random sampling strategy based on the *t*-distribution. Γ denotes the Gamma function, while σ denotes the mutation scaling factor. This strategy enables broader exploration of the search space during the early iterations, while favoring more focused local search in the later stages.

#### 3.1.4. Operations for the Selfing Phase

During the selfing phase, restorer individuals are updated. When an individual has reached the maximum selfing limit SC but fails to update the global optimal individual, this suggests that the algorithm has converged to a local optimum, thus requiring a reset.

This paper refines SC by allowing it to vary adaptively throughout the iterative process, as depicted in Equation (22).

(22) $$SC={\mathrm{SC}}_{\min}+\left({\mathrm{SC}}_{\max}-{\mathrm{SC}}_{\min}\right)\cdot \left(1-{\left(\frac{t}{T}\right)}^{2}\right).$$

During the algorithm’s initialization and global exploration, most individuals are highly likely to make productive moves within a short iteration cycle. Accordingly, the SC parameter is initialized with a comparatively high value. When an individual meets this criterion, this suggests premature convergence and triggers a reset. As the search advances, the chance of stagnation in local optima grows; so, a smaller SC is used to accelerate escape from such regions.

In addition, this paper also improves the selfing strategy for restorer individuals. The gene update formula for restorer individuals is replaced by Equations (23)–(25),

(23) $$x_{r(i)}\left(t+1\right)=r_{3}\cdot \left(X_{\mathrm{best}}-X_{r(j)}+c_{1}\cdot \left(LB+L\right)\right),$$

(24) L=α·Lévy(β)·(UB−LB),

(25) $$c_{1}=2\cdot \exp\left(-{\left(\frac{4t}{T}\right)}^{2}\right),$$

where α serves as the step-length scaling coefficient, and $c_{1}$ is an adaptively varying factor that gradually decreases from 2 toward 0 in a nonlinear manner as the iteration proceeds. $x_{r(j)}$ denotes a restorer individual randomly chosen from the population (j≠i), while $r_{3}$ is a uniformly distributed random variable within [0, 1]. The term Lévy(*β*) follows a Lévy flight distribution characterized by the parameter β whose heavy-tailed nature allows the algorithm to perform frequent local movements interspersed with occasional long jumps, thereby improving both global exploration and local refinement capabilities [42]. The explicit mathematical form of this distribution is presented in Equation (26).

(26) $$Lévy\left(\beta \right)∼t^{-\mu },\;1<\beta \leq 3.$$

**Figure 3:**
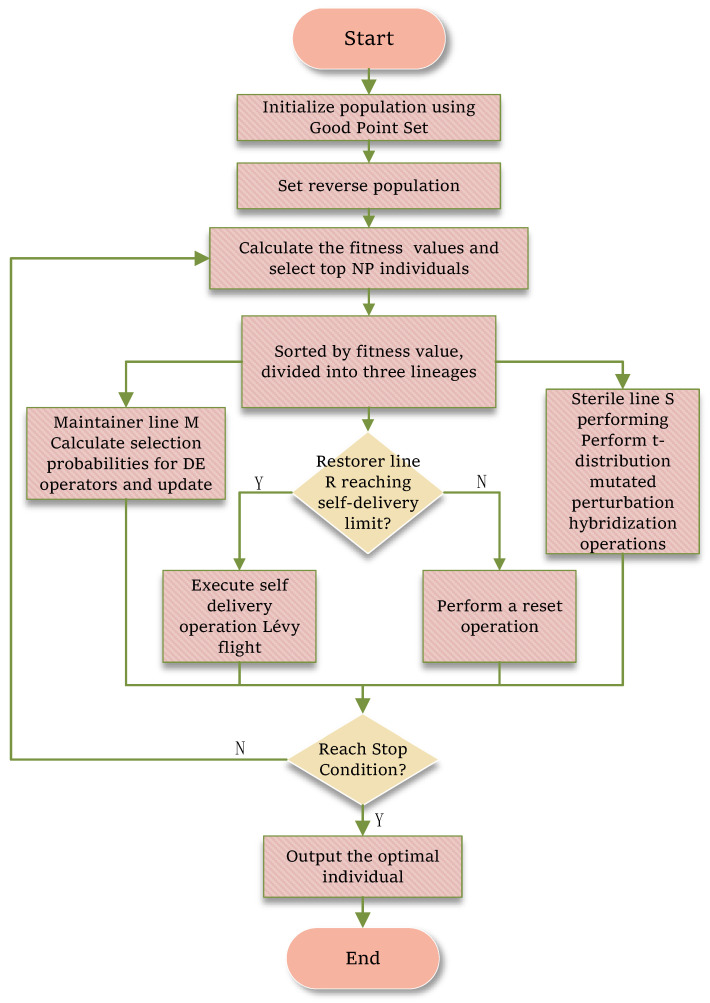
The flowchart of the IHBOFS algorithm.

The flowchart of the IHBOFS is shown in Figure 3.

### 3.2. IHBOFS Method

The proposed feature selection method, based on IHBOFS, follows the workflow outlined below. Initially, the algorithm parameters are set, and the search space is defined according to the feature count in the dataset. Next, cross-validation is applied to partition the data. IHBOFS is then utilized on the training set to identify the optimal solution. Although HBO is designed for continuous space optimization, FS is inherently a discrete problem. Thus, a binary encoding technique is employed to adapt the original algorithm for continuous domains to a form that can address discrete challenges. To evaluate the performance of the selected feature subset, an SVM classifier is used, and a fitness function is calculated to assess the subset’s quality by balancing the classification accuracy and feature minimization.

#### 3.2.1. Binary Encoding

Through experimental comparison among eight transfer functions, we selected the most suitable one for the current scenario, as shown in Equation (27):

(27) $$S\left(x\right)=\frac{1}{1+\exp(-x/2)}.$$

The continuous values are mapped into the [0,1] interval through the transfer function. Then, the transformed value is converted into a binary solution representation using Equation (28):

(28) $$X_{i}=\left\{\begin{array}{cc} 1, & \mathrm{if}\;T(X_{i})>\mathrm{rand}\\ 0, & \mathrm{otherwise}. \end{array}\right.$$

Here, “1” indicates that the corresponding feature is selected for classifier training, while “0” means the feature is not selected. T(·) denotes the transfer function, and rand is a random value within the range [0,1].

#### 3.2.2. Fitness Function

The fitness function in feature selection must balance two conflicting objectives: classification performance and subset dimensionality. Relying solely on accuracy tends to produce oversized feature subsets, whereas minimizing the number of features alone often results in severe information loss and degraded predictive performance. To select as few features as possible while improving the accuracy, the fitness function designed in this paper is defined as follows:

(29) $$\mathrm{Fitness}=\lambda \cdot \left(1-\mathrm{acc}\right)+\mu \cdot \frac{n}{N},$$

where Fitness represents the fitness value, acc is the classification accuracy, and *n* and *N* represent the proportion of selected features to the total number of features, λ+μ=1. The term (1−acc) ensures that maximizing the accuracy corresponds to minimizing the fitness value, which aligns with the minimization nature of evolutionary optimization. The ratio $\frac{n}{N}$ provides a scale-invariant measure of subset size, preventing bias toward datasets with different dimensionalities.

#### 3.2.3. Complexity Analysis

With *N* as the population size, *D* as the dimension of the search space, and *T* as the iteration limit, the computational complexity for each operational stage is presented in Table 2.

Even though the time complexity for initializing the population in IHBOFS is roughly twice that of HBO, its effect on the overall time complexity remains minimal, as the population initialization occurs only once. IHBOFS incurs a moderate increase in time complexity, approximately one-third, due to the addition of a preservation sub-population update. However, this enhancement significantly improves the exploitation of high-quality individuals and enhances the population diversity. Notably, the time complexities of the population sorting and optimal solution updates remain consistent between IHBOFS and HBO. Therefore, the proposed improvement strategy maintains a relatively low additional computational cost while enhancing the algorithm’s search performance and population diversity, making it a relatively excellent improvement strategy.

## 4. Experiment Results and Discussion

The effectiveness of HBO has been demonstrated in function optimization and engineering problems. As a result, this paper concentrates on feature selection experiments, and three groups of experiments are organized:

1. Ablation Study: this analysis aims to evaluate the role and significance of each component within the proposed IHBOFS, and experiments are conducted using different combinations of strategies on the CEC2022 test functions.
2. Classifier and Transfer Function Selection: using high-dimensional real datasets from the Scikit feature selection repository, six classifiers (KNN, SVM, XGBoost, Decision Tree, Random Forest, Naive Bayes) are evaluated and selected based on performance; in addition, both standard HBO and IHBOFS are combined with eight different transfer functions to perform feature selection, in order to identify the best classifier and transfer function.
3. Comparison with Other Algorithms: based on the selected SVM classifier and the S3 transfer function, IHBOFS is compared with various feature selection algorithms based on metaheuristics across multiple high-dimensional datasets.

All algorithms in the experiments are implemented in Python 3.8.20. Experiments are conducted on a computer equipped with an AMD Ryzen 7 5800H CPU @ 3.2 GHz and 32 GB RAM, using Windows 10 as the operating system.

### 4.1. Datasets

The CEC2022 benchmark suite includes 1 unimodal function (F1), 4 basic multimodal functions (F2–F5), 3 hybrid functions (F6–F8), and 4 composite functions (F9–F12), with a dimensionality of 20. A detailed description is provided in Appendix A.

The high-dimensional datasets are obtained from the standard Scikit feature selection database, as shown in Table 3. These datasets contain between 1024 and 10,304 features and 100 to 400 samples. All are multi-class classification problems, each presenting unique challenges. Their diversity provides a robust basis for evaluating IHBOFS’s ability to identify optimal feature subsets across various complex scenarios.

### 4.2. Experimental Settings

Table 4 presents the key parameter settings of HBO and its four improved strategy variants. For equitable evaluation and to confirm the contribution of each enhancement mechanism, each algorithm is independently executed 30 times on each test function at different dimensions. The maximum number of iterations is set to 1000 for all algorithms.

To ensure fairness in the number of fitness evaluations, the population size is set to 40 for IHBOFS and HBO_DE and to 60 for standard HBO and the other three improved algorithms. Five evaluation metrics are employed to comprehensively assess the algorithms’ performance: best fitness value (Best), worst fitness value (Worst), mean fitness value (Mean), standard deviation of fitness values (Std), and average execution time (Time).

To further assess the performance of IHBOFS, it is compared with several metaheuristic-based algorithms, including GA, Flower Pollination Algorithm (FPA) [43], Sparrow Search Algorithm (SSA) [44], Whale Optimization Algorithm (WOA) [45], the Rime-Ice Optimization Algorithm (RIME) [46], JAYA [47], GWO, and Harris Hawk Optimization (HHO) [48]. The parameter settings of these algorithms are listed in Table 5. All algorithms use a population size of 40 and a maximum of 1000 iterations. Each test function is run independently 30 times to minimize the impact of randomness. The parameter settings and evaluation metrics of HBO and IHBOFS are consistent with those used in the ablation study.

In the classifier evaluation and selection experiment, all classifiers are independently run 10 times on each dataset. A five-fold cross-validation and stratified sampling strategy is employed to reduce overfitting.

Properly designed transfer mechanisms can improve the interpretability and controllability of the feature selection process [49]. This study selects eight transfer functions (four S-shaped and four V-shaped), whose details are provided in Appendix B.

### 4.3. Comparison of Experimental Results and Analysis

Detailed experimental results and analyses are presented in the following subsections.

#### 4.3.1. Ablation Study

Based on the results shown in Table 6, integrating all four improvement strategies into IHBOFS yields clear and consistent advantages over both the original HBO and all individually enhanced variants. IHBOFS achieves superior performance in terms of both the Best and Mean metrics, while further reducing the standard deviation, demonstrating significantly enhanced stability across multiple runs. Overall, the integrated strategy effectively leverages the complementary strengths of each individual enhancement, resulting in an optimized exploration process across the three populations and substantially improving the algorithm’s overall problem-solving performance and robustness.

#### 4.3.2. Classifier Performance Evaluation and Selection

Ten independent runs were conducted for each classifier–dataset combination to ensure the statistical robustness of the validation findings. A five-fold cross-validation combined with stratified sampling was applied to mitigate overfitting risks. Table 7 summarizes the average classification accuracy and training time over 10 runs for several classifiers, including KNN, SVM, XGBoost, DT, RF, and NB.

As shown in Table 7, SVM exhibits strong overall performance with an average accuracy of 83.70%, ranking second only to RF, which attained 83.86%. Notably, SVM achieved exceptional accuracy on the pixraw10P and orlraws10P datasets, reaching 97.00% and 96.00% respectively, significantly outperforming other classifiers. This highlights the superior ability of SVM to handle high-dimensional data. In contrast, NB performs poorly on certain datasets such as pixraw10P, with an accuracy of only 22.00%, indicating that it may not be suitable for specific scenarios. Moreover, NB exhibits limited generalization across datasets, with its accuracy consistently lagging behind that of SVM and RF.

**Figure 4:**
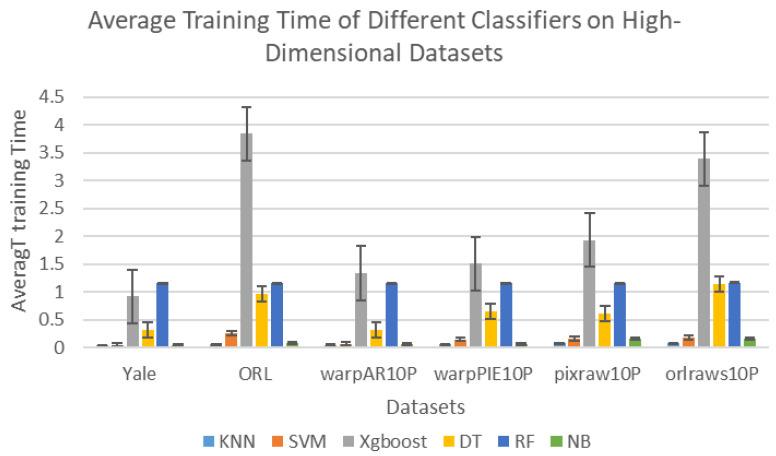
Average Training Time of Different Classifiers on High-Dimensional Datasets.

To provide a more intuitive comparison, Figure 4 presents the bar chart of training times for different classifiers. From the training time perspective, KNN is the fastest among all classifiers, significantly outperforming the others. SVM combines high accuracy with favorable training speed, averaging 0.145 s per run. This is 87.52% faster than RF (1.1617 s on average) and also substantially lower than XGBoost (2.155 s). This advantage is particularly important in real-time or resource-constrained environments.

In conclusion, SVM demonstrates dual strengths in classification accuracy and operational efficiency. Although RF offers a slight edge in accuracy, its longer training time may become a constraint in practical applications. Therefore, for high-dimensional classification tasks where both speed and accuracy are critical, KNN and SVM are generally more effective options. This study will further evaluate the classification performance of KNN and SVM under different parameter settings.

This study primarily focuses on the dimensionality reduction capability of evolutionary algorithms and their discriminative power across diverse categories. Given the complexity of parameter combinations across different classifiers, only key parameters significantly impacting classification performance are locally tuned. This approach emphasizes the search performance and efficiency of the evolutionary algorithm rather than the classifier itself.

**Figure 5:**
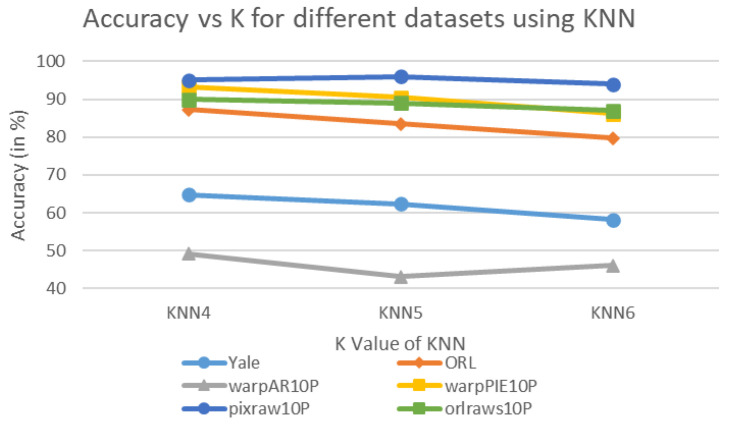
KNN classification accuracy under different *K* values.

**Figure 6:**
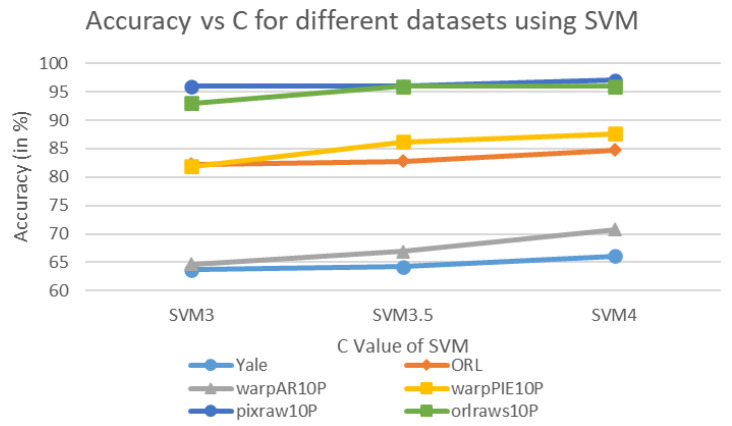
SVM classification accuracy under different *C* values.

Figure 5 and Figure 6 illustrate the classification accuracy of KNN and SVM under different parameter settings. Specifically, for KNN, the number of neighbors *K* is tested at values of 4, 5, and 6. For SVM, the penalty parameter *C* is evaluated at 3, 3.5, and 4. These parameters strongly influence the model’s performance and fitting behavior. In KNN, *K* determines the number of neighbors considered during classification, affecting the smoothness and generalization. For SVM, *C* controls the decision boundary margin and the tolerance to misclassifications, thereby determining the model complexity. As observed from Figure 5, as the value of *K* increases, KNN’s accuracy tends to decrease across most datasets. This suggests that a smaller *K* may provide more concentrated information and thus higher accuracy. On the other hand, SVM exhibits improved performance as *C* increases. Notably, SVM with C=4 (SVM4) shows superior accuracy on multiple datasets, especially on pixraw10P and orlraws10P, where it achieves 97.00% and 96.00%, respectively.

To prevent potential overfitting and to ensure generalization across datasets, this study adopts cross-validation and stratified sampling. Both strategies help reduce bias in the train–test split and provide a more reliable estimate of model performance. Moreover, since *C* in SVM determines the model complexity, a larger value may increase the risk of overfitting. However, the results in Table 7 show that using smaller *C* values does not improve the performance; instead, the accuracy decreases. Objectively, if the model were overfitting at C=4, reducing the value of *C* (e.g., to 3 or 3.5) would simplify the model and alleviate overfitting, thereby improving the accuracy. However, the opposite trend is observed, indicating that the model is not overfitting at C=4. Although larger *C* values may potentially yield higher accuracy, they also tend to enforce stricter fitting to the training samples, which could overshadow the feature-filtering capability of the metaheuristic algorithm. Therefore, excessively large *C* values are not further explored in this study.

With regard to the training time, the table shows that KNN and SVM demonstrate stable training times across different parameter settings. Overall, SVM4 exhibits a favorable trade-off, especially on complex and high-dimensional datasets. Its high accuracy and stability highlight strong modeling capability. Moreover, SVM shows better generalization than KNN across datasets. A reasonable training time further ensures its practicality. Based on these findings, SVM with C=4 is selected as the final classifier configuration for the subsequent experiments.

#### 4.3.3. Transfer Function Experiments

To identify the most suitable transfer function for the algorithm, this subsection evaluates the performance of the multi-strategy improved IHBOFS in combination with the eight transfer functions introduced in Section 4.2. Feature selection experiments are conducted using the optimal classifier (SVM) selected in the previous subsection. Table 8 presents the classification accuracy of IHBOFS when combined with different transfer functions. In the table, Best denotes the highest accuracy among 10 independent runs, Worst denotes the lowest accuracy, while Mean and Std represent the average accuracy and standard deviation, respectively.

According to the results, the feature subsets obtained using S-shaped transfer functions significantly outperform those obtained using V-shaped functions. Among all transfer functions, S3 yields the best average classification performance when combined with IHBOFS, achieving the highest mean accuracy on five out of the tested datasets.

The combinations IHBOFS+S1, IHBOFS+S2, and IHBOFS+S3 all achieve the highest Best score on the last three high-dimensional datasets. Notably, S3 further enhances the lower-bound performance of IHBOFS, as evidenced by its superior Worst scores compared to other transfer functions. As a result, IHBOFS+S3 outperforms all other combinations in terms of overall average accuracy.

For the first three datasets, IHBOFS+S3 performs best across almost all accuracy metrics. Although IHBOFS+S3 does not achieve the highest average accuracy on the warpAR10P dataset (87.69% for IHBOFS+S4), the difference between the two is less than 1.79%. Overall, S3 produces feature subsets with better classification performance.

Table 9 records the number of features selected by IHBOFS with different transfer functions. Although V-shaped functions sometimes select fewer features on certain datasets, their classification accuracy is significantly inferior to that of S-shaped functions. Therefore, when classification performance is prioritized, V-shaped functions are not suitable as the final choice.

Moreover, the IHBOFS+S3 combination achieves the lowest standard deviation on three datasets, indicating enhanced stability. It also demonstrates the ability to select smaller feature subsets with higher classification accuracy, especially on the latter three high-dimensional datasets, outperforming other S-shaped functions.

#### 4.3.4. Comparison with Other Algorithms and Result Analysis

In the previous sections, we compared the performance of different classifiers and transfer functions and selected SVM and the S3 transfer function as the final settings for the classification model. Based on this configuration, this section compares IHBOFS with various metaheuristic-based feature selection algorithms, including HHO, the Ant Colony Optimization Algorithm (ACO) [50], Slime Mould Algorithm (SMA) [51], Artificial Bee Colony Algorithm (ABC) [52], Tree Growth Algorithm (TGA) [53], Henry Gas Solubility Optimization Algorithm (HGSO) [54], Emperor Penguin Optimizer Algorithm (EPO) [55], Manta Ray Foraging Optimization Algorithm (MRFO) [56], and the standard HBO.

As shown in Table 10, IHBOFS achieves higher feature subset accuracy compared to all other algorithms. The only exception is the pixraw10P dataset, where IHBOFS ranks second to MRFO in terms of the mean classification accuracy. In all other datasets, IHBOFS outperforms the other algorithms across Best, Worst, and Mean metrics.

In particular, HGSO produces relatively weak feature subsets. On the Yale dataset, IHBOFS surpasses HGSO by 14.65% in mean classification accuracy and by 13.19% and 8.70% on warpAR10P and warpPIE10P, respectively. It is worth emphasizing that, while the original HBO performs better than most algorithms on the first four datasets, it fails to effectively search for optimal feature subsets on the two higher-dimensional datasets—an issue addressed by the improved IHBOFS.

In terms of the number of selected features and runtime, the EPO achieves the best performance. However, the EPO tends to prioritize selecting fewer features while compromising the classification performance. For instance, on the Yale dataset, the EPO’s Best and Mean scores are 73.33% and 72.06%, respectively—10.75% and 11.19% lower than IHBOFS.

**Figure 7:**
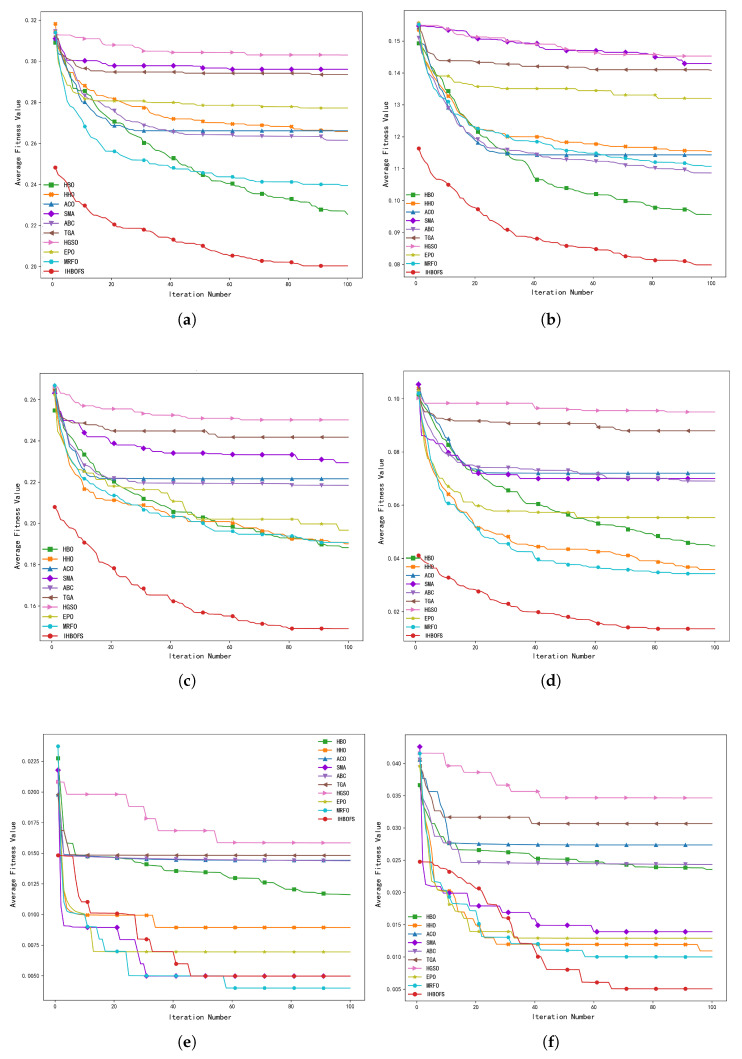
Average convergence curves of comparison algorithms on high-dimensional datasets. (**a**) Yale. (**b**) ORL. (**c**) warpAR10P. (**d**) warpPIE10P. (**e**) pixraw10P. (**f**) orlraws10P.

Figure 7 presents the convergence curves of the average fitness values for all comparison algorithms. As shown, IHBOFS only ranks second to MRFO on the pixraw10P dataset. For all other datasets, IHBOFS achieves the smallest fitness values, significantly outperforming other algorithms. Moreover, IHBOFS exhibits better initial fitness values, thanks to the use of the Good Point Set and Elite Opposition-Based Learning strategies for population initialization, which helps generate higher-quality candidate solutions and accelerates convergence toward the global optimum.

**Figure 8:**
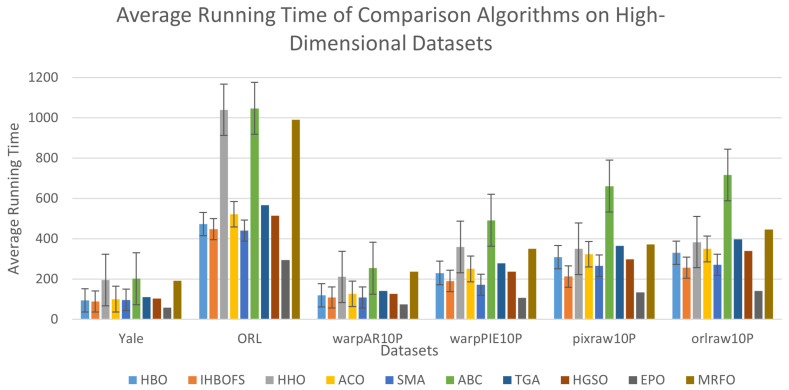
Average running time of comparison algorithms on high-dimensional datasets.

Figure 8 visually compares the runtime of different algorithms. Although the EPO has the shortest runtime, IHBOFS—despite integrating multiple advanced strategies—does not exhibit performance degradation compared to HBO. Instead, it shows improvement, as IHBOFS selects fewer and higher-quality feature subsets, thus accelerating the search process. On higher-dimensional datasets, IHBOFS also demonstrates higher efficiency compared to other algorithms, confirming its suitability for high-dimensional feature selection tasks.

Table 11 shows the algorithm ranking obtained using the Friedman non-parametric statistical test based on mean accuracy. A lower rank indicates better performance. As shown, IHBOFS achieves the best average rank of 1.1667, followed by MRFO. TGA and HGSO rank lowest, indicating their inability to identify optimal feature subsets in this context. The computed *p*-value is significantly lower than the standard significance threshold (0.05), supporting the conclusion that performance differences among the algorithms are statistically significant.

## 5. Conclusions

In this paper, an adaptive improvement scheme for the Hybrid Breeding Optimization algorithm is proposed to address the problem of high-dimensional feature selection. The main contributions of this study are summarized as follows: First, the performance bottlenecks of HBO in high-dimensional and complex optimization tasks are analyzed, and IHBOFS is developed. IHBOFS incorporates several innovative strategies in a targeted manner, and these improvements comprehensively enhance the search ability and robustness of the algorithm at different evolutionary stages. Ablation experiments are conducted on 12 benchmark functions of different types and dimensions, and IHBOFS achieves the optimal average performance on 10 of these functions with significant reductions in the objective function values. In addition, IHBOFS exhibits zero or near-zero variance on several functions, reflecting extremely strong stability.

For high-dimensional feature selection tasks, a wrapper framework based on IHBOFS is designed in this paper, which maps continuous search spaces to discrete ones by means of multiple transfer functions. Combined with classifier selection and local fine-tuning strategies, this framework further improves the quality of the selected feature subsets and simultaneously enhances the classification performance. Extensive experimental validations show that IHBOFS achieves the highest mean classification accuracy on all six datasets, with the average accuracy improvement ranging from 2.54% to 3.49% compared with the original HBO, and its performance on each dataset outperforms other state-of-the-art metaheuristic algorithms. In terms of solution robustness, IHBOFS also has lower or comparable standard deviations to the comparison algorithms, indicating more stable search behavior. Furthermore, the improvements in IHBOFS lead to a maximum reduction of 98.8% and 94.0% in the size of the selected feature subsets while still maintaining extremely high accuracy. IHBOFS also demonstrates competitiveness in terms of the computational time.

Despite the promising results achieved above, several directions are worthy of further exploration. First, future research can incorporate the stability of feature subsets into the design of the fitness function to realize the collaborative optimization of classification accuracy, subset size, and stability. Second, considering the challenge of label scarcity in practical applications, future work can explore semi-supervised or unsupervised feature selection methods to better utilize unlabeled or partially labeled data, thereby improving the adaptability and practical application value of the algorithm.

## Figures and Tables

**Table 1:** Summary of nascent hybrid algorithms for feature selection.

Algorithm	Domain	Performance Metrics	Limitations
ISPSO [32]	High-dimensional feature selection, classification tasks	Accuracy reaches 93.5%, with redundant features reduced by approximately 12%.	Sensitive to initial parameter selection; requires high adaptability to specific high-dimensional scenarios; susceptible to feature dimensionality.
Dynamic PSO [33]	Feature selection for dynamic data streams	Accuracy ranges from 88% to 92%; capable of handling high-dimensional dynamic data.	Suitable for dynamic environments but struggles with real-time high-frequency changes; high computational resources.
SU-GA [34]	Feature selection for bioinformatics data	Average accuracy attains 91%.	Poor performance on small-scale datasets.
PSO-GWO [35]	Various domains in UCI repositories, hybrid feature selection	Average accuracy of 90% with reduced computation time.	Further research needed on alternative fitness and transfer functions.
Hybrid Ant Lion PSO [36]	Feature identification for gene expression data	Prediction accuracy of 87.88%.	Room for improvement in convergence rate for large-scale data processing.
EWOA [37]	Internet of Things (IoT)	Average accuracy of 95.5% on Aalto IoT dataset and 98.8% on RT-IoT 2022 dataset; average feature reduction rate of 82.5% (Aalto IoT) and 62.3% (RT-IoT 2022).	Prone to the curse of dimensionality due to initial parameters; excellent performance on static tasks but weak adaptability to dynamic data.
GOG-MBSHO [38]	Cancer gene feature selection, high-dimensional classification	Outperforms all comparative algorithms in average fitness value across selected datasets; average number of selected features is 63.	Risk of performance degradation when processing extremely large datasets.
I-KOA [39]	Liver disease classification, medical data feature selection	Overall classification accuracy of 93.46%; feature selection size of 0.1042.	High computational cost and insufficient algorithm adaptability.
HFCCW [40]	Network anomaly detection	Accuracy of 92%; feature selection rate ranges from 10% to 20%.	High computational time consumption.

**Table 2:** Time complexity comparison analysis.

Stage	HBO	IHBOFS
Population Initialization	O(N)	O(2N)
Preservation Sub-population Update	-	$O\left(\frac{N\cdot D}{3}\right)$
Crossover	$O\left(\frac{N\cdot D}{3}\right)$	$O\left(\frac{N\cdot D}{3}\right)$
Self-crossover	$O\left(\frac{N\cdot D}{3}\right)$	$O\left(\frac{N\cdot D}{3}\right)$
Population Sorting	O(N)	O(N)
Optimal Solution Update	O(1)	O(1)
Total	$O\left(T\cdot (N+2N\cdot D/3+1)+N\right)$	$O\left(T\cdot (N+N\cdot D+1)+2N\right)$

**Table 3:** Dataset description.

ID	Dataset	Number of Samples	Number of Features	Number of Classes
D1	Yale	165	1024	15
D2	ORL	400	1024	10
D3	warpAR10P	130	2400	40
D4	warpPIE10P	210	2400	10
D5	pixraw10P	100	10,000	10
D6	orlraws10P	100	10,304	10

**Table 4:** Parameter settings for different improved strategies.

Algorithms	Parameter Setting
HBO	SC=8
HBO_INIT	SC=8, s∈[0.01,0.92]
HBO_DE	F=0.5, cr=0.9, SC=8
HBO_TD	σ∈[0.1,1.0], gw=2, SC=8
HBO_SC	SC∈[4,10]

**Table 5:** Key parameter settings for comparison algorithms.

Algorithms	Parameter Settings
IHBOFS	$F=0.5,cr=0.9,g_{r}=2,a=0.1,$
	s∈[0.01,0.92],σ∈[0.10,1.0],
	${\mathrm{SC}}_{\max}=10,{\mathrm{SC}}_{\min}=4$
GA	CR=0.8, MR=0.01
FPA	p=0.8, β=1.5
SSA	$c_{1},c_{2}\in \left[0,1\right]$
WOA	*b* = 1
RIME	*W* = 1.5
JAYA	−
GWO	α∈[0,2]
HHO	β = 1.5

**Table 6:** Ablation study results of the improvement strategies.

Function	HBO	HBO_INIT	HBO_DE	HBO_TD	HBO_SC	IHBOFS
	Mean ± Std	Mean ± Std	Mean ± Std	Mean ± Std	Mean ± Std	Mean ± Std
F1	310.55 ± 17.63	317.80 ± 47.96	316.91 ± 31.64	307.11 ± 13.78	4787.90 ± 1189.40	300.00 ± 0.00
F2	465.03 ± 9.89	461.10 ± 10.63	459.11 ± 14.66	485.41 ± 19.14	588.89 ± 30.81	448.94 ± 0.75
F3	600.00 ± 0.00	600.00 ± 0.00	600.00 ± 0.01	600.00 ± 0.00	600.00 ± 0.00	600.00 ± 0.00
F4	930.62 ± 34.88	927.49 ± 26.82	914.28 ± 46.02	1048.30 ± 20.70	982.61 ± 31.13	878.03 ± 28.94
F5	901.13 ± 0.61	900.23 ± 0.26	900.49 ± 0.35	902.36 ± 0.70	901.45 ± 0.64	900.24 ± 0.20
F6	707,110 ± 565,160	1,445,900 ± 2,195,100	34,424 ± 12,929	23,070,000 ± 15,035,000	631,090 ± 991,950	45,885 ± 13,119
F7	1951.40 ± 83.46	1980.70 ± 79.73	1955.40 ± 81.69	2192.60 ± 247.34	2067.40 ± 51.13	1987.40 ± 76.82
F8	2887.40 ± 510.22	2984.00 ± 583.25	2303.90 ± 166.67	6223.30 ± 2264.10	3241.90 ± 352.97	2154.00 ± 101.03
F9	2642.60 ± 7.06	2628.80 ± 61.09	2624.70 ± 60.27	2639.30 ± 2.23	2741.20 ± 123.17	2635.70 ± 0.05
F10	2358.00 ± 887.07	2144.30 ± 1011.40	2480.90 ± 678.15	2577.20 ± 512.51	2847.50 ± 481.64	2527.60 ± 370.76
F11	2613.90 ± 11.51	2612.50 ± 11.26	2615.60 ± 18.51	2623.30 ± 3.04	2609.50 ± 11.71	2600.00 ± 0.01
F12	2939.40 ± 18.22	2942.70 ± 20.43	2927.80 ± 9.85	2929.40 ± 7.68	3068.50 ± 55.48	2900.00 ± 0.00

**Table 7:** Classification accuracy (%) and training time of different classifiers.

Classifiers	Yale		ORL		warpAR10P		warpPIE10P		pixraw10P		orlraws10P		Average	
	ACC	TIME	ACC	TIME	ACC	TIME	ACC	TIME	ACC	TIME	ACC	TIME	ACC	TIME
KNN	64.85	**0.04**	87.25	0.06	49.23	**0.05**	**93.33**	**0.06**	95.00	**0.08**	90.00	**0.08**	79.94	**0.0617**
SVM	66.06	0.05	84.75	0.26	70.77	0.07	87.62	0.15	**97.00**	0.16	96.00	0.18	83.70	0.1450
Xgboost	60.61	0.92	75.00	3.84	68.46	1.34	84.76	1.51	80.00	1.93	72.00	3.39	73.47	2.1550
DT	49.52	0.31	23.40	0.97	69.54	0.32	69.29	0.65	89.60	0.61	74.10	1.14	62.58	0.6667
RF	**70.12**	1.16	75.90	1.16	**78.54**	1.16	86.48	1.16	95.80	1.16	**96.30**	1.17	**83.86**	1.1617
NB	63.64	0.05	**88.25**	0.09	59.23	0.06	87.14	0.07	22.00	0.16	93.00	0.16	68.88	0.0983
KNN4	64.85	**0.04**	**87.25**	**0.06**	49.23	**0.05**	**93.33**	**0.06**	95.00	**0.08**	90.00	**0.08**	79.94	**0.0617**
KNN5	62.42	**0.04**	83.50	**0.06**	43.08	**0.05**	90.48	**0.06**	96.00	**0.08**	89.00	**0.08**	77.41	**0.0617**
KNN6	58.18	**0.04**	79.75	0.07	46.15	**0.05**	86.19	0.07	94.00	**0.08**	87.00	**0.08**	75.21	0.0650
SVM3	63.64	0.05	82.25	0.27	64.62	0.07	81.90	0.15	96.00	0.16	93.00	0.18	80.24	0.1467
SVM3.5	64.24	0.05	82.75	0.26	66.92	0.07	86.19	0.15	96.00	0.17	**96.00**	0.18	82.02	0.1467
SVM4	**66.06**	0.05	84.75	0.26	70.77	0.07	87.62	0.15	**97.00**	0.16	**96.00**	0.18	**83.70**	0.1450

**Table 8:** Classification accuracy (%) of IHBOFS with different transfer functions.

Dataset	Measures (%)	S1	S2	S3	S4	V1	V2	V3	V4
Yale	Best	80.00	80.00	**81.21**	80.00	79.39	80.00	79.39	78.79
	Worst	78.18	78.18	**78.79**	78.18	75.76	75.76	76.36	75.15
	Mean	79.33	79.27	**80.12**	79.45	77.45	77.27	77.58	77.03
	Std	0.57	**0.45**	0.59	0.57	1.11	1.09	1.05	1.26
ORL	Best	**93.00**	92.00	**93.00**	**93.00**	91.50	91.00	92.00	91.25
	Worst	90.75	90.50	91.75	**91.25**	89.75	89.55	89.75	89.75
	Mean	91.33	91.25	92.35	**92.23**	90.73	90.25	90.50	90.30
	Std	0.63	**0.40**	0.44	0.61	0.57	0.50	0.72	0.46
warpAR10P	Best	85.38	86.92	86.15	**87.69**	84.62	84.62	86.92	83.85
	Worst	81.54	82.31	**83.85**	82.31	80.77	79.23	80.77	80.77
	Mean	84.15	84.23	**85.15**	84.38	82.15	82.38	83.08	82.38
	Std	1.10	1.34	**0.60**	1.62	1.13	1.52	1.65	1.11
warpPIE10P	Best	**100.00**	**100.00**	**100.00**	99.52	99.05	99.52	99.52	98.10
	Worst	97.14	97.14	**97.62**	97.14	95.71	95.24	96.67	94.76
	Mean	98.52	98.38	**98.81**	98.52	97.05	97.48	97.71	96.52
	Std	**0.86**	**0.86**	**0.86**	0.69	1.22	1.51	0.92	1.04
pixraw10P	Best	**100.00**	**100.00**	**100.00**	**100.00**	**100.00**	**100.00**	**100.00**	**100.00**
	Worst	**99.00**	**99.00**	**99.00**	**99.00**	**99.00**	**99.00**	**99.00**	**99.00**
	Mean	99.40	**99.60**	99.50	**99.60**	99.50	**99.60**	99.40	**99.60**
	Std	**0.49**	**0.49**	0.50	**0.49**	0.50	**0.49**	**0.49**	**0.49**
orlraws10P	Best	**100.00**	**100.00**	**100.00**	**100.00**	**100.00**	99.00	**100.00**	**100.00**
	Worst	98.00	**99.00**	**99.00**	98.00	**99.00**	98.00	98.00	98.00
	Mean	99.50	99.50	99.50	99.20	99.30	**98.80**	99.00	99.00
	Std	0.81	0.50	0.50	0.60	0.46	**0.40**	0.45	0.63

**Table 9:** Number of selected features by IHBOFS with different transfer functions.

Transfer Functions	Yale		ORL		warpAR10P		warpPIE10P		pixraw10P		orlraws10P	
	Avg	Std	Avg	Std	Avg	Std	Avg	Std	Avg	Std	Avg	Std
S1	409.10	**31.49**	410.60	50.08	454.10	176.20	430.20	159.87	24.00	9.86	138.90	188.01
S2	397.60	48.38	451.00	42.01	474.90	233.89	430.00	205.92	27.90	13.49	**91.00**	**38.04**
S3	361.70	77.88	420.70	**25.98**	472.00	**131.48**	253.60	117.91	**20.00**	**5.39**	110.40	68.14
S4	408.40	51.10	**364.50**	70.05	419.30	202.34	351.20	155.36	21.30	6.07	154.90	119.42
V1	**262.60**	59.37	373.10	68.78	400.40	208.35	430.10	264.03	39.30	15.32	123.70	55.84
V2	287.30	66.58	408.70	69.11	458.80	204.12	332.40	261.49	45.10	28.07	132.70	53.86
V3	290.20	80.84	401.40	50.58	**386.50**	137.02	**218.90**	**93.76**	40.30	31.87	151.10	92.95
V4	313.10	71.76	405.90	50.04	417.60	141.61	418.30	232.10	41.80	26.16	155.40	105.44

**Table 10:** Experimental results of comparison algorithms on high-dimensional datasets.

Datasets	Algorithms	Best	Worst	Mean	Std	Num	Time
D1	HBO	78.79	76.36	77.58	0.61	351.6	94.47
	IHBOFS	**81.21**	**78.79**	**80.12**	0.59	361.70	88.69
	HHO	75.76	71.52	73.39	1.34	238.30	194.68
	ACO	75.76	72.12	73.58	1.09	464.80	100.05
	SMA	71.52	69.09	70.18	0.76	89.60	96.89
	ABC	75.15	71.52	74.00	1.03	418.70	201.66
	TGA	72.12	69.70	70.85	0.69	504.80	110.49
	HGSO	70.30	69.09	69.88	**0.39**	493.20	103.12
	EPO	73.33	70.91	72.06	0.88	**63.60**	**58.53**
	MRFO	77.58	72.12	76.06	1.52	246.60	192.29
D2	HBO	91.50	89.75	90.72	0.55	384.20	472.91
	IHBOFS	**93.00**	**91.75**	**92.35**	0.44	420.70	447.11
	HHO	90.50	87.50	88.63	1.13	281.90	1040.11
	ACO	90.25	87.50	88.92	0.74	479.00	521.61
	SMA	87.50	85.00	85.67	0.68	113.10	440.60
	ABC	89.75	89.25	89.47	**0.21**	457.90	1046.83
	TGA	87.00	85.75	86.28	0.36	503.40	567.56
	HGSO	86.75	85.25	85.83	0.45	506.40	514.93
	EPO	87.75	85.25	86.78	0.79	**112.90**	**293.91**
	MRFO	91.50	88.00	89.15	0.97	339.20	990.85
D3	HBO	83.85	80.00	81.31	1.09	751.60	119.10
	IHBOFS	**86.15**	**83.85**	**85.15**	0.60	472.00	108.14
	HHO	83.08	78.46	80.92	1.75	280.10	210.61
	ACO	79.23	76.92	78.08	0.71	1093.70	126.27
	SMA	80.00	75.38	76.85	1.21	41.90	108.36
	ABC	79.23	77.69	78.38	**0.41**	1059.10	253.99
	TGA	76.92	74.62	76.08	0.64	1186.90	141.09
	HGSO	76.92	73.85	75.23	0.83	1190.60	127.22
	EPO	83.08	76.15	80.15	2.25	**34.00**	**74.33**
	MRFO	83.08	79.23	80.92	1.23	432.90	237.55
D4	HBO	98.10	94.29	95.81	1.04	765.30	230.18
	IHBOFS	**100.00**	**97.62**	**98.81**	0.86	253.60	190.31
	HHO	97.62	95.71	96.43	0.65	115.10	359.06
	ACO	94.29	91.90	93.19	0.74	1109.20	250.45
	SMA	94.76	90.95	92.95	1.12	45.40	171.51
	ABC	94.76	92.86	93.48	0.71	1078.30	491.03
	TGA	92.38	90.95	91.62	0.49	1203.90	277.40
	HGSO	91.43	90.48	90.09	**0.26**	1203.20	236.71
	EPO	95.71	93.33	94.43	0.80	**40.60**	**107.09**
	MRFO	99.05	92.86	96.67	1.92	313.90	350.61
D5	HBO	99.00	**99.00**	99.00	**0.00**	1716.90	308.34
	IHBOFS	**100.00**	**99.00**	99.50	0.50	20.00	212.40
	HHO	**100.00**	**99.00**	99.10	0.30	23.00	350.00
	ACO	99.00	**99.00**	99.00	**0.00**	4523.60	322.90
	SMA	**100.00**	**99.00**	99.50	0.50	16.90	266.08
	ABC	99.00	**99.00**	99.00	**0.00**	4480.80	661.02
	TGA	99.00	**99.00**	99.00	**0.00**	4917.50	365.06
	HGSO	99.00	98.00	98.90	0.30	4958.10	298.39
	EPO	**100.00**	**99.00**	99.30	0.46	**13.60**	**134.40**
	MRFO	**100.00**	**99.00**	**99.60**	0.49	31.50	372.00
D6	HBO	98.00	97.00	97.80	0.40	1830.80	330.51
	IHBOFS	**100.00**	**99.00**	**99.50**	0.50	110.40	256.00
	HHO	**100.00**	98.00	98.90	0.70	47.30	383.50
	ACO	98.00	97.00	97.70	0.46	4730.30	349.58
	SMA	100.00	98.00	98.60	0.66	45.20	270.70
	ABC	98.00	98.00	98.00	**0.00**	4670.80	716.17
	TGA	98.00	97.00	97.40	0.49	5080.50	397.87
	HGSO	97.00	97.00	97.00	**0.00**	5107.60	338.67
	EPO	**100.00**	98.00	98.70	0.90	**28.40**	**140.43**
	MRFO	**100.00**	98.00	99.00	0.77	110.20	445.54

**Table 11:** Friedman test for the average accuracy of comparison algorithms.

Algorithm	Average Ranking	Final Rank	*p*-Value
IHBOFS	1.166667	1	8.182420 × 10^−10^
MRFO	2.666667	2	
HBO	3.833333	3	
HHO	4.333333	4	
EPO	5.333333	5	
ABC	5.500000	6	
ACO	6.500000	7	
SMA	7.166667	8	
TGA	8.666667	9	
HGSO	9.833333	10	

## Data Availability

All datasets employed in this research were obtained from the open-access UCI Machine Learning Repository (https://archive.ics.uci.edu/ (accessed on 15 March 2025)).

## Appendix A. Description of the CEC2022 Benchmark Functions

The CEC2022 test suite is one of the latest benchmark suites widely used for evaluating the performance of optimization algorithms. It consists of 12 single-objective test functions with boundary constraints, covering four types: unimodal functions, multimodal functions, hybrid functions, and composite functions. The design objectives of these four categories of functions are described as follows:

Unimodal Functions
  Unimodal functions have only one global optimal solution and no other local optimal solutions in the search space. This category of functions is mainly used to test the convergence speed and accuracy of optimization algorithms, which is a core indicator to measure the basic optimization capability of algorithms.
Multimodal Functions
  Multimodal functions possess multiple local optimal solutions and one global optimal solution in the search space. They are designed to evaluate the global search capability of optimization algorithms and their ability to avoid falling into local optima. To achieve better performance on multimodal functions, algorithms need to balance exploration and exploitation in the search process, where exploration focuses on discovering new search regions, and exploitation refines the current promising regions.
Hybrid Functions
  Hybrid functions are usually composed of multiple basic test functions, integrating the characteristics of both unimodal and multimodal functions to create more complex and challenging optimization problems. Such functions may contain both easily optimizable regions and difficult-to-optimize regions simultaneously, requiring algorithms to have the ability to switch search strategies adaptively and perform effective searches in different types of search spaces.
Composition Functions
  Composition functions further extend the concept of hybrid functions by combining multiple hybrid functions or basic functions to construct extremely complex test environments. These functions typically have higher dimensions and more intricate search spaces. The core objective of composite functions is to provide a broader range of challenges to assess the adaptability and robustness of algorithms when facing various types of optimization problems, while also requiring algorithms to have stronger global optimization capabilities and higher computational efficiency to handle the increased complexity.

All benchmark functions share the same *D*-dimensional global search range:

$$x_{i}\in \left[-100,100\right],\;i=1,\ldots ,D.$$

Table A1 summarizes the 12 benchmark functions used in this study, including their IDs, types, names, and global optimum values.

**Table A1:** CEC2022 benchmark functions used in this study.

Type	ID	Function Name	Optimal Value
Unimodal Function	F1	Shifted and Full Rotated Zakharov Function	300
Multimodal Functions	F2	Shifted and Full Rotated Rosenbrock’s Function	400
	F3	Shifted and Full Rotated Expanded Schaffer’s f6 Function	600
	F4	Shifted and Full Rotated Non-Continuous Rastrigin’s Function	800
	F5	Shifted and Full Rotated Levy Function	900
Hybrid Functions	F6	Hybrid Function 1 (N = 3)	1800
	F7	Hybrid Function 2 (N = 6)	2000
	F8	Hybrid Function 3 (N = 5)	2200
Composition Functions	F9	Composition Function 1 (N = 3)	2300
	F10	Composition Function 2 (N = 4)	2400
	F11	Composition Function 3 (N = 5)	2600
	F12	Composition Function 4 (N = 6)	2700

Global search range: ${\left[-100,100\right]}^{D}$.

Unimodal Function (F1): tests global convergence capability without complex local minima.

Multimodal Functions (F2–F5): evaluate the ability to escape local minima while maintaining exploration.

Hybrid Functions (F6–F8): divide the decision vector into segments, each associated with different basic optimization functions.

Composition Functions (F9–F12): represent the most challenging landscapes by combining several base functions through weighted adaptive composition strategies.

## Appendix B. Transfer Functions in Feature Selection

The feature selection problem is often regarded as a complex combinatorial optimization challenge. However, many metaheuristic algorithms, originally designed for continuous space problems, cannot be directly applied to discrete optimization problems such as feature selection. Therefore, how to effectively convert the search in the continuous space into decisions in the discrete space is a key challenge. Transfer functions act as a bridge in this conversion process: through transfer functions, algorithms can search freely in the continuous domain, while their solutions can be interpreted as the selection or exclusion of features.

Transfer functions allow the search capabilities and flexibility of metaheuristic algorithms to be preserved, enabling the algorithms to perform excellently on global optimization problems. They can be applied to various types of feature selection tasks, whether for text, image, or bioinformatics data. By rationally designing the conversion mechanism, the interpretability and controllability of the feature selection process can be improved. In this paper, eight transfer functions (four sigmoid-type and four V-type) are selected; detailed information on these transfer functions is shown in Table A2. Transfer functions ensure the consistency of the search agents’ positions in the continuous and discrete spaces. Specifically, sigmoid-type transfer functions tend to map large positive values in the continuous space to 1 with a high probability; in contrast, V-type transfer functions tend to map values with large absolute values in the continuous space to 1 with a high probability.

**Table A2:** Details of the S-shaped and V-shaped transfer functions.

S-Shaped Functions		V-Shaped Functions	
Function Symbol	Transfer Function	Function Symbol	Transfer Function
$S_{1}$	$S\left(x\right)=\frac{1}{1+exp(-2x)}$	$V_{1}$	$V\left(x\right)=\left|\frac{2}{\pi }\arctan\left(\frac{\pi x}{2}\right)\right|$
$S_{2}$	$S\left(x\right)=\frac{1}{1+exp(-x)}$	$V_{2}$	$V\left(x\right)=\left|\frac{x}{\sqrt{1+x^{2}}}\right|$
$S_{3}$	$S\left(x\right)=\frac{1}{1+exp(-x/2)}$	$V_{3}$	$V\left(x\right)=\left|\tanh(x)\right|$
$S_{4}$	$S\left(x\right)=\frac{1}{1+exp(-x/3)}$	$V_{4}$	$V\left(x\right)=\left|\frac{\sqrt{2}}{\pi }\int_{0}^{\sqrt{\pi }/2x}exp\left(-s^{2}\right)ds\right|$
